# Comparative evaluation of dermatoglyphic patterns between skeletal class I and skeletal class III malocclusion

**DOI:** 10.12688/f1000research.127895.1

**Published:** 2023-01-10

**Authors:** Sonika Achalli, U S Krishna Nayak, Murali P S, Keerthan Shashidhar, Vinayak Kamath

**Affiliations:** 1 Oral Medicine and Radiology, Nitte Deemed to be University, AB Shetty Memorial Institute of Dental Sciences, Mangalore, Karnataka, 575018, India; 2 Orthodontics and Dentofacial Orthopaedics, Nitte Deemed to be University, AB Shetty Memorial Institute of Dental Sciences, Mangalore, Karnataka, 575018, India; 3 Public Health Dentistry, Goa Dental College and Hospital, Bambolim, North Goa, Goa, 403202, India

**Keywords:** dermatoglyphics, skeletal malocclusion, fingerprint, ridge count

## Abstract

**Background:** Dermatoglyphics is the study of various dermal configurations on the fingers, palms, and soles. These appear during the 12th week of intrauterine life and develop completely by the 24th week. It is said that they remain constant thereafter. The aim of the present study was to compare and assess the association of dermatoglyphic patterns between skeletal class I and skeletal class III malocclusion.

**Methods:** Finger and palm prints were collected using the ink and roller method from 604 subjects who were divided into skeletal class I, class III with maxillary retrognathism and class III with mandibular prognathism based on lateral cephalogram assessment.

**Results:** Loop pattern was more predominant in skeletal class I malocclusion subjects and whorl pattern was more frequent in the other two groups. Total finger ridge count and atd angle also showed significant difference between the study groups.

**Conclusions:** The present study attempted in assessing the association between dermatoglyphic patterns and skeletal malocclusion. Analysing dermal configurations may aid in indicating the type of developing malocclusion and thus help in interceptive and preventive orthodontics.

## Introduction

The study of dermal ridge counts and patterns on the fingers, palms and soles is referred to as ‘dermatoglyphics.’ ^1^ Cummins and Midlo coined the term dermatoglyphics in the year 1962 ^2^ and defined it as the study of complex dermal ridge configurations on the skin covering the palmar and plantar surfaces of the hands and feet. It has been stated that studying the various dermal patterns on the fingers, palms and soles can help diagnose many diseases; mostly caused due to chromosomal abnormalities. ^3^ The term dermatoglyphics has been derived from two Greek words; derma meaning skin and glyhe meaning carve. ^4^

Literature says that the 12 ^th^ week of intra-uterine life is characterized by the appearance of the dermal configurations and is completely established by the 24 ^th^ week. ^5^ It is believed that the configurations remain the same after that, except for the change in size. The teeth, alveolus and the palate develop during the same time as the dermal configurations, hence it is reported that there is an association between the two. ^5^ Dermatoglyphics was initially studied in Down’s syndrome where abnormal dermatoglyphic patterns were noted. Later, various other medical conditions like Klinefelter syndrome, Turner’s syndrome, rubella syndrome, and leukaemia also have shown to exhibit abnormal or unusual dermal patterns. ^6^ Expression of the gene is said to be the basis for the craniofacial growth and hence is accountable for skeletal malocclusions. Alterations in dermatoglyphic configurations might be a reflection of genetic or chromosomal abnormalities and hence may be used to study genetically influenced diseases. ^7^ The dermal configurations are said to be unique for a particular individual; hence, studying these may help diagnose and treat certain genetic disorders in the individuals examined. ^8^

**Figure 1.**
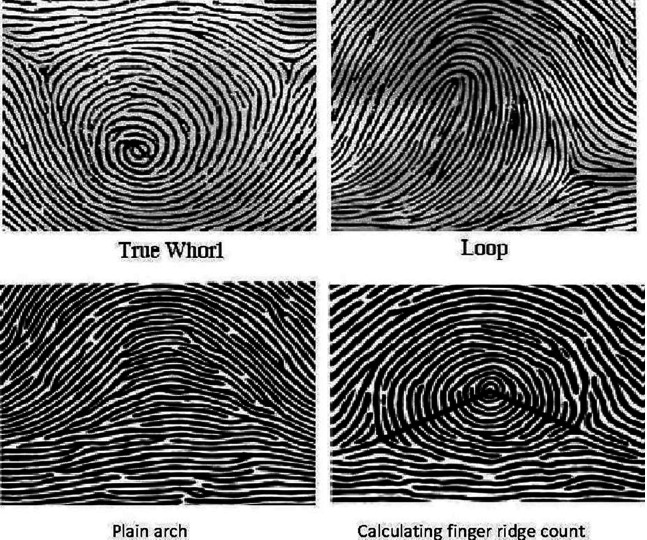
Dermatoglyphic patterns and calculation of finger ridge count.

**Figure 2.**
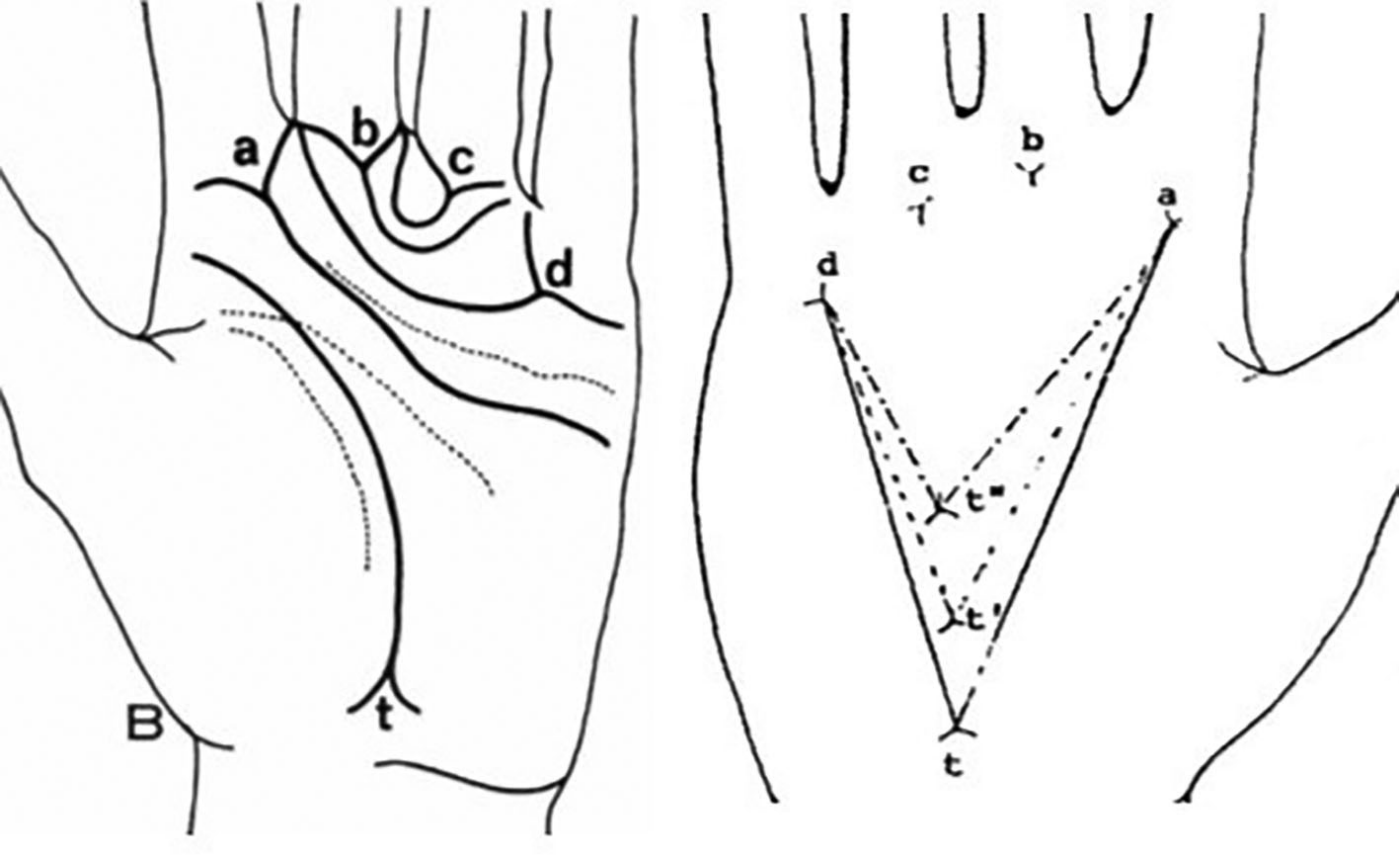
atd angle; ‘a, b, c, d’ are digital triradius and ‘t’ is axial triradius.

The basic classification of the dermal patterns was given by Sir Francis Dalton in 1892. ^9^ The fingerprint patterns have been mainly classified into three types i.e., arches, loops, and whorls. There may be two types of arches: simple or tented; two types of loops which may be ulnar or radial depending upon the direction they face and three types of whorls: symmetrical, spiral, and double loop ( Figure 1). ^1^^,^^3^ The atd angle is presented in the whole palm and this is formed by straight lines drawn from the digital triradius ‘a’ to the axial triradius ‘t’ and from this triradius to the digital triradius ‘d’ ( Figure 2). ^3^^,^^10^

In the present study we have evaluated the fingerprint patterns, total finger ridge count (TFRC) and atd angle and correlated them with skeletal malocclusion.

## Methods

A convenience sample of 604 subjects including males and females reporting to the outpatient departments of Oral Medicine and Orthodontics, A B Shetty Memorial Institute of Dental Sciences, Karnataka, India were selected for the present observational study. Based on the previous studies; ^7^ considering the effect size of 0.13, alpha error 5% and beta error 20%, a sample size of 192 was determined which was rounded off to 200 subjects per group. During the course of the study, 201 subjects in group I and II and 202 subjects in group III were included. The subjects were recruited between the period of September 2016 to January 2021. The subjects were aged between 21 and 30 years. Group I comprised of subjects with class I skeletal malocclusion where their SNA (sella tursica-nasion-point A) was 82±2 degrees and SNB (sella tursica-nasion-point B) was 80±2 degrees. Group II included subjects with class III skeletal malocclusion with maxillary retrognathism where SNA was less than 80 degrees and SNB was 80±2 degrees. Group III included subjects with skeletal malocclusion with mandibular prognathism where their SNB was more than 82 degrees with SNA 82±2 degrees. Cephalometric analysis was done by an experienced orthodontist and dermatoglyphic analysis was done by the principal investigator in order to avoid any bias. A pilot study was done initially with a sample size of 30 subjects in each group assessing the relationship between only fingerprint patterns and skeletal malocclusion. ^11^

Subjects with any one of the following conditions were excluded: malformation syndromes associated with maxilla and mandible; facial asymmetry and acquired skeletal defects; history of trauma/injury or surgical procedures done in the orofacial region; history of habits like mouth breathing, thumb sucking, lip biting, tongue thrusting; malformations of the fingers and palms which is congenital or acquired, amputated fingers, skin diseases, and wound or scars on the fingers.

Subjects were informed about the method and procedure of the study following which written informed consent was obtained. Ethical approval was received from the central ethics committee of the university (NU/CEC/2016-2017/0078 dated 12/08/2016). Lateral cephalogram was assessed to identify the type of skeletal malocclusion. The finger and palm prints of both hands were recorded by ink and roller method as described by Cummins and Midlo. ^2^ First, the subjects were asked to wash their hands with soap and water in order to remove any oily secretions, dirt and sweat and then dried with a towel. A small amount of ink was applied on the inking slab and a thin and even film of ink was obtained by rolling it thoroughly onto the entire surface of the slab. The palmar surface of the right hand was placed on the inking slab with a gentle press. The inked palmar surface was then gently pressed onto a clean, white bond paper and removed. The procedure was repeated for the left hand. In order to take the rolled impressions of the individual fingers, the bulb of the finger was placed at right angle to the surface of the inked slab and then rolled or turned until the bulb faced the opposite direction. The finger was then placed onto a clean, white bond paper and rolled in a similar manner. A clean, rolled impression of the fingerprint was obtained. ^2^

Ridge count was calculated by drawing a line from the core to the triradius and counting the number of ridges the line touches or crosses ( Figure 1) in each digit. The arch pattern has no triradius, there is one triradius in loop pattern and the whorl pattern has two triradii. ^3^

**Figure 3.**
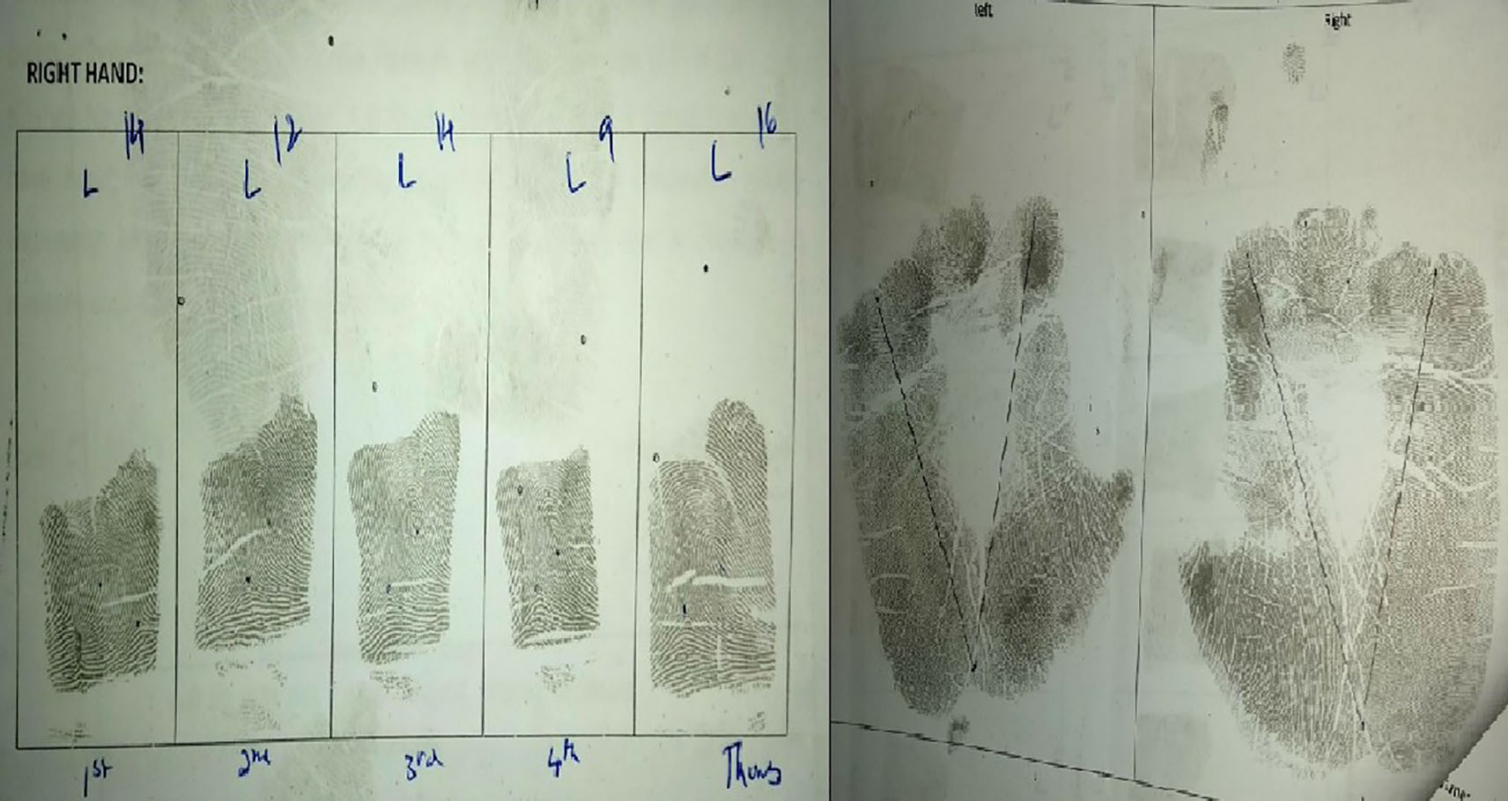
Finger and palm print with finger ridge count and atd angle.

A palm print is required to measure the atd angle. Atd angle was estimated as an angle formed by joining the lines drawn from the digital triradius ‘a’ to the axial triradius ‘t’ and from this triradius to another digital triradius ‘d’ ( Figure 2). ^10^ Data was thus collected and analysed for various fingerprint patterns, total finger ridge count and atd angle of both hands ( Figure 3).

The obtained data was then statistically analysed. The data was analysed using Statistical Package for Social Sciences software version 22 (IBM SPSS Statistics, Armonk, NY: IBM Corp). The distribution of different dermatoglyphic configurations on each hand in each class of malocclusion was calculated using percentages. The association between class of malocclusion and the dermatoglyphic pattern was tested using Fisher’s Exact test where p<0.05 was considered significant. Comparison of the variables between study groups was done using chi-squared and Kruskal-Wallis tests where p<0.05 was considered significant.

## Results

The study sample comprised of 604 subjects with 201 subjects in in group I and II; 202 subjects in group III aged between 21 and 30 years. ^12^ Group I had 67 males and 134 females with a mean age of 24.02±2.99 years, group II had 109 males and 92 females with a mean age of 23.78±2.27 years and group III had 105 males and 97 females with a mean age of 24.09±2.57 years.

The obtained data was then statistically analysed. The distribution of different dermatoglyphic configurations on each hand in each class of malocclusion was calculated using percentages. The association between class of malocclusion and the dermatoglyphic pattern was tested using Fisher’s Exact test where p<0.05 was considered significant ( Table 1). Loop pattern was predominant in group I subjects (i.e., class I skeletal malocclusion) and whorl pattern occurred more frequently in group II (i.e., class III skeletal malocclusion where maxilla is retrognathic) and group III subjects (i.e., class III skeletal malocclusion where mandible is prognathic). The frequencies of the dermatoglyphic patterns differed significantly between group I and the other two study groups but there was no significant difference between group II and group III subjects ( Table 1).

**Table 1.** Distribution of study participants according to RT (right) and LT (left) hand pattern.

Hand pattern		Group			Fisher’s Exact Test
		Group I	Group II	Group III	p-value
**RT hand pattern**	**A**	0	1	0	<0.001 *
		0.0%	0.5%	0.0%	
	**L**	145	59	62	
		72.1%	29.4%	30.7%	
	**W**	56	141	140	
		27.9%	70.1%	69.3%	
**LT hand pattern**	**A**	0	1	0	<0.001 *
		0.0%	0.5%	0.0%	
	**L**	151	60	63	
		75.1%	29.9%	31.2%	
	**W**	50	140	139	
		24.9%	69.7%	68.8%	

* Results are statistically significant where p<0.05.

The total finger ridge count in each hand was calculated and the mean value was determined. The atd angles of the right and left palm were also determined. Comparison of these variables between study groups was done using appropriate statistical tests (chi-squared, Kruskal-Wallis tests) where p<0.05 was considered significant ( Table 2). TFRC differed significantly between group I and other two study groups whereas no significant difference was seen between group II and group III subjects. Mean TFRCs of both hands were the highest in group I subjects and lower in group II and group III subjects. Similarly, atd angle also differed significantly between group I and other two study groups with no significant difference between group II and group III ( Table 2).

**Table 2.** Comparison of variables between the study groups. [RT FRC=right finger ridge count; LT FRC=left finger ridge count].

Variables	Group	N	Mean	SD	Min	Max	Percentiles			Kruskal-Wallis Test	
							Q1	Median	Q3	Chi-squared value	p-value
**RT FRC**	**Group I**	201	40.67	21.27	0	72	27	43	59.5	272.19	<0.001 *
	**Group II**	201	12.29	18.97	0	59	0	0	26.5		
	**Group III**	202	12.42	19.15	0	57	0	0	34.5		
**LT FRC**	**Group I**	201	38.96	22.01	0	72	20	41	57	250.33	<0.001 *
	**Group II**	201	12.64	19.55	0	56	0	0	27		
	**Group III**	202	12.45	19.12	0	55	0	0	32.25		
**RT ATD**	**Group I**	201	40.59	5.37	33	64	38	40	42.5	323.44	<0.001 *
	**Group II**	201	28.11	9.57	20	55	21	23	39		
	**Group III**	202	27.71	9.00	20	49	21	23	38.25		
**LT ATD**	**Group I**	201	40.95	4.78	33	54	38	41	43	316	<0.001 *
	**Group II**	201	28.17	9.93	20	54	21	23	40		
	**Group III**	202	27.70	8.95	20	46	21	23	39		

* Results are statistically significant where p<0.05.

## Discussion

Depending on the genetic background, the effect of an active environmental factor on a particular phenotype varies; this in turn will affect the structure developing during the same time. ^7^^,^^13^ The facial structures and the dermal ridges of the fingers and palm develop from the ectoderm. Structures of the craniofacial complex and the epidermal ridges form during the same time i.e., second trimester of the intrauterine life. It has been deciphered that any hereditary or environmental factors causing various malocclusions may also trigger abnormalities in dermatoglyphic patterns. ^7^^,^^13^ The study of dermal ridge configurations of the fingers, palms and soles can be a predominant diagnostic tool for many disorders particularly those with genetic abnormalities which may be associated with the deformation of fingerprint pattern. ^3^ Hence, the present study was undertaken to evaluate and correlate the various dermatoglyphic patterns with skeletal class I and skeletal class III malocclusion.

### Fingerprint pattern

In the present study, loop pattern was more predominant in both hands of group I subjects with skeletal class I malocclusion and higher frequencies of whorl pattern was observed in both hands of group II (subjects with skeletal class III malocclusion with maxillary retrognathism) and group III subjects (subjects with skeletal class III malocclusion with mandibular prognathism). The results were statistically significant.

Loop pattern was more frequent in the skeletal class I malocclusion group in the studies done by Eslami N *et al.* ^14^ and Gautham N *et al.* ^15^ The present study also showed similar results. In the study conducted by Charles A *et al.* ^16^ loop pattern was predominant in both skeletal class I and class III malocclusion. Some studies have contrasting results like in studies conducted by Reddy BRM *et al.*, ^7^ Jindal G *et al.* ^17^ and Tikare S *et al.* ^18^ Nonsignificant results were obtained by Reddy BRM *et al.* ^7^ pertaining to the class I malocclusion group, but a higher frequency of whorls was seen in class II and class III malocclusion. Jindal G *et al.* ^17^ found in their study that dermatoglyphic pattern was not specific to any particular class of malocclusion, although higher frequency of whorls was seen in class II and class III malocclusion. The study by Tikare S *et al.* ^18^ showed that the frequency of whorl pattern was equally distributed in all three classes of malocclusion. The contrast in results could be because in the above studies Angle’s classification of malocclusion was taken to classify the different malocclusion groups whereas in the present study skeletal malocclusion was considered.

### Total finger ridge count (TFRC)

The total finger ridge count of both hands was calculated. The present study showed that mean TFRCs were the lower in group II and group III subjects and highest in group I subjects. The results were statistically significant between group I and the other two study groups. This was in accordance with the studies done by Jindal G *et al.* ^17^ and Eslami N *et al.* ^14^ The study by Reddy BRM *et al.* ^7^ showed an increase in TFRCs in all groups of malocclusions with no statistical significance. The variation in results in the present study could be due to the reason that Angle’s classification of malocclusion (molar relationship) was taken into consideration for grouping the subjects whereas in the present study skeletal malocclusion was considered.

### Atd angle

Palm prints of both hands were taken from all subjects to measure the atd angle. Mean atd angle was found to be higher in group I subjects when compared to the other two study groups. The results were statistically significant. The study done by Jindal G *et al.* ^17^ also showed a significant difference in atd angle among the different groups of malocclusions. Other studies have shown varying results. The study done by Reddy BRM *et al.* ^7^ showed that the mean atd angle was higher in the study groups when compared to the control group i.e., class I occlusion. The study done by Eslami N *et al.* ^14^ showed no significant difference in mean atd angle between Angle’s skeletal class I, class II and class III subjects. The reason for the varying results could be because Angle’s classification (dental) was used to classify malocclusion whereas in the present study skeletal malocclusion was considered.

In the current study, racial, ethnic variations and hereditary factors were not considered which could be included in future studies. Recent advances like digital finger and palmprint method may be used instead of the ink and roller method used in the present study. Further, whether it can be relied as a sole factor for prediction of skeletal malocclusion is still questionable.

## Conclusions

The current study strived to evaluate and correlate the various dermatoglyphic patterns with skeletal malocclusion. The results showed that a particular type of malocclusion may be specific to a certain dermatoglyphic pattern. Loop pattern was more predominant in skeletal class I malocclusion subjects and whorl pattern was more predominant in skeletal class III subjects. Mean TFRCs and mean atd angle was higher in skeletal class I malocclusion subjects when compared to skeletal class III subjects. Based on the results obtained in the present study, dermatoglyphic patterns may be used as an indicator of skeletal malocclusion. This shows that it could be used as a diagnostic tool at an early age with a fair degree of accuracy and may aid in intercepting and preventing the developing skeletal malocclusion.

## Data Availability

Figshare: data sheet 1.xlsx. https://doi.org/10.6084/m9.figshare.21550662. ^12^ This project contains the following underlying data:
• data sheet 1.xlsx (Data sheet showing the results obtained during the study) Data are available under the terms of the Creative Commons Attribution 4.0 International license (CC-BY 4.0).
