# Supplementary material for: Comparative evaluation of dermatoglyphic patterns between skeletal class I and skeletal class III malocclusion
Source: F1000Res. 2023 Apr 3;12:37. Originally published 2023 Jan 10. [Version 2] doi: 10.12688/f1000research.127895.2 (PMC10806363; doi:10.12688/f1000research.127895.2)
Supplement: Peer review history

# Reviewer report: Reviewer response for version 2

**DOI:** 10.5256/f1000research.146358.r209108

**Published:** 2024-01-23

**Authors:** Gowri Sankar Singaraju

**Affiliations:** 1 Department of Orthodontics, Narayana Dental College, Nellore, Andhra Pradesh, India

**Title**: Comparative evaluation of dermatoglyphic patterns between skeletal class I and skeletal class III malocclusion --- **A Cross Sectional Study. **Capitalize each word in the Title. Indicate the type of study in the Title.

**Abstract **- Methods: Make grouping clear: **Group I - class I; Group II - class III with maxillary retrognathism; Group III - class III **with mandibular prognathism based on lateral cephalogram assessment.

**Abstract - **Results **: **if analytical methods used, please indicate them. It seems some comparative and associative analysis was done.

**Introduction: **The purpose of the study is well established

**Methods: **Very neatly explained. Include the details of ethical committee certificate if possible in the beginning itself. It is mentioned that a pilot study was done. Does the final sample include the sample for pilot study also?

 State the reason why excess sample has to be taken such as say 10 or 20 percent above the calculated sample size.

**Results:** it seems that KW test was found to be statistically significant as shown in table 2, your writing part indicates that you have done intrapair comparisons probably by Mann Whitney test. If possible include it in the table. Mention this in statistical analysis

Is the work clearly and accurately presented and does it cite the current literature?

Yes

If applicable, is the statistical analysis and its interpretation appropriate?

Yes

Are all the source data underlying the results available to ensure full reproducibility?

Yes

Is the study design appropriate and is the work technically sound?

Yes

Are the conclusions drawn adequately supported by the results?

Yes

Are sufficient details of methods and analysis provided to allow replication by others?

Yes

Reviewer Expertise:

Dental- Orthodontics

I confirm that I have read this submission and believe that I have an appropriate level of expertise to confirm that it is of an acceptable scientific standard.

# Reviewer report: Reviewer response for version 2

**DOI:** 10.5256/f1000research.146358.r168606

**Published:** 2023-04-12

**Authors:** Preethi Balan

**Affiliations:** 1 Singapore Oral Microbiomics Initiative, National Dental Research Institute Singapore, National Dental Center, Singapore, Singapore

I am satisfied with the changes made by the authors. I am agreeable to the acceptance of this manuscript.

Is the work clearly and accurately presented and does it cite the current literature?

Yes

If applicable, is the statistical analysis and its interpretation appropriate?

I cannot comment. A qualified statistician is required.

Are all the source data underlying the results available to ensure full reproducibility?

Yes

Is the study design appropriate and is the work technically sound?

Yes

Are the conclusions drawn adequately supported by the results?

Yes

Are sufficient details of methods and analysis provided to allow replication by others?

Partly

Reviewer Expertise:

NA

I confirm that I have read this submission and believe that I have an appropriate level of expertise to confirm that it is of an acceptable scientific standard.

# Reviewer report: Reviewer response for version 1

**DOI:** 10.5256/f1000research.140441.r159764

**Published:** 2023-01-26

**Authors:** Preethi Balan

**Affiliations:** 1 Singapore Oral Microbiomics Initiative, National Dental Research Institute Singapore, National Dental Center, Singapore, Singapore

In this study, the authors aimed to determine the association between dermatoglyphic patterns and skeletal malocclusion. The authors observed a predominance of loop patterns in skeletal class I and whorl patterns in skeletal class III. Total finger ridge count and atd angle showed significant differences between the study groups. The study has clinical relevance in terms of early identification and intervention of malocclusion. Please see below few suggestions.

1. Please provide the full form of atd in the abstract.
2. Please include values of statistical interpretation in the abstract.
3. Please cite the reason for rounding off the sample size to 200 after sample size estimation.
4. The authors mention that a pilot study was done initially with a sample size of 30 subjects. Was the data from the pilot study incorporated into the existing study?
5. “Cephalometric analysis was done by an experienced orthodontist, and dermatoglyphic analysis was done by the principal investigator in order to avoid any bias”- Please remove the term principal investigator and mention the initials of the researcher who performed the analysis.
6. Also, please mention who performed the data collection on fingerprint patterns, TFRC and atd angle. Was any calibration exercise performed before data collection?
7. Please cite the criteria for the cephalometric classification of patients in this study.
8. “During the course of the study, 201 subjects in group I and II and 202 subjects in group III were included”. Please rewrite this sentence for clarity in the methods and results section; Groups should be distinctly described.
9. Please include full forms of abbreviations at the first mention in the text-

- TFRC in the abstract and main text
- Provide legends for abbreviated terms in Table 1
- 10. Please cite or obtain permission to publish if Fig 1 and 2 are not from the existing original research

Is the work clearly and accurately presented and does it cite the current literature?

Yes

If applicable, is the statistical analysis and its interpretation appropriate?

I cannot comment. A qualified statistician is required.

Are all the source data underlying the results available to ensure full reproducibility?

Yes

Is the study design appropriate and is the work technically sound?

Yes

Are the conclusions drawn adequately supported by the results?

Yes

Are sufficient details of methods and analysis provided to allow replication by others?

Partly

Reviewer Expertise:

Oral health research

I confirm that I have read this submission and believe that I have an appropriate level of expertise to confirm that it is of an acceptable scientific standard, however I have significant reservations, as outlined above.

# Response

**Published:** 2023-03-30

**Authors:** Sonika Achalli

**Affiliations:** Nitte Deemed to be University, AB Shetty Memorial Institute of Dental Sciences, India

Dear Dr Preethi Balan, 

 Thank you for reviewing our article and giving us your expert suggestions.

- **Reviewer 1**: *Please provide the full form of atd in the abstract.*
**Comments from authors:** Suggestion has been incorporated
- **Reviewer 1**: *Please include values of statistical interpretation in the abstract.*
**Comments from authors:** Suggestion has been incorporated
- **Reviewer 1**: *Please cite the reason for rounding off the sample size to 200 after sample size estimation.*
**Comments from authors:** Citation for the sample size calculation has been added. As suggested by the statistician the sample size was rounded off for calculation purpose.
- **Reviewer 1:** *The authors mention that a pilot study was done initially with a sample size of 30 subjects. Was the data from the pilot study incorporated into the existing study?*
**Comments from authors**: Yes, the data from the pilot study has been incorporated into the existing study
- **Reviewer 1:** *“Cephalometric analysis was done by an experienced orthodontist, and dermatoglyphic analysis was done by the principal investigator in order to avoid any bias”- Please remove the term principal investigator and mention the initials of the researcher who performed the analysis.*
**Comments from authors:** Suggestion has been incorporated
- **Reviewer 1:** *Also, please mention who performed the data collection on fingerprint patterns, TFRC and atd angle. Was any calibration exercise performed before data collection?*
**Comments from authors:** Calibration exercise was not done but single examiner i.e principal investigator has performed the data collection and this has now been incorporated.
- **Reviewer 1:** *Please cite the criteria for the cephalometric classification of patients in this study.*
**Comments from authors:** Suggestion has been incorporated
- **Reviewer 1:** *“During the course of the study, 201 subjects in group I and II and 202 subjects in group III were included”. Please rewrite this sentence for clarity in the methods and results section; Groups should be distinctly described.*
**Comments from authors:** Suggestion has been incorporated
- **Reviewer 1**: *Please include full forms of abbreviations at the first mention in the text-*
  - *TFRC in the abstract and main text*
  - *Provide legends for abbreviated terms in Table 1*
- **Comments from authors: **Suggestion incorporated
- **Reviewer 1:** *Please cite or obtain permission to publish if Fig 1 and 2 are not from the existing original research*
**Comments from authors:** Suggestion incorporated
